# Liver myofibroblasts from hepatitis B related liver failure patients may regulate natural killer cell function via PGE2

**DOI:** 10.1186/s12967-014-0308-9

**Published:** 2014-11-04

**Authors:** Min Zhang, Fenglan Wang, Yutian Chong, Qiang Tai, Qiyi Zhao, Yubao Zheng, Liang Peng, Shumei Lin, Zhiliang Gao

**Affiliations:** Department of Infectious Diseases, Third Affiliated Hospital of Sun Yat-sen University, No. 600 Tianhe Road, Guangzhou, 510630 Guangdong Province China; Department of Infectious Diseases, First Affiliated Hospital of Medical College of Xi′an Jiaotong University, No. 277 Yanta West Road, Xi′an, 710061 Shanxi Province China; Department of Infectious Diseases, the Eighth Hospital of Xi′an, Xi′an, China; Department of Hepatic Surgury, First Affiliated Hospital of Sun Yat-sen University, Guangzhou, China

**Keywords:** Liver myofibroblasts, Natural killer cell, Immune-mediated liver injury, Liver failure, Hepatitis B

## Abstract

**Background:**

Natural killer (NK) cells are abundant in the liver and constitute a major innate immune component that contributes to immune-mediated liver injury. However, few studies have investigated the phenotypes and functions of NK cells involved in hepatitis B related liver failure (LF), and the precise mechanism underlying NK cell regulation is not fully understood.

**Methods:**

We detected the percentage and function of peripheral NK cells both in hepatitis B related LF patients and healthy volunteers by flow cytometry and isolated the liver myofibroblasts (LMFs) from hepatitis B related LF livers. To determine the possible effects of LMFs on NK cells, mixed cell cultures were established in vitro.

**Results:**

We found a down-regulated percentage of peripheral NK cells in hepatitis B related LF patients, and their NK cells also displayed decreased activated natural cytotoxicity receptors (NCRs) and cytokine production. In a co-culture model, LMFs sharply attenuated IL-2-induced NK cell triggering receptors, cytotoxicity, and cytokine production. The inhibitory effect of LMFs on NK cells correlated with their ability to produce prostaglandin (PG) E2.

**Conclusion:**

These data suggest that LMFs may protect against immune-mediated liver injury in hepatitis B related LF patients by inhibiting NK cell function via PGE2.

**Electronic supplementary material:**

The online version of this article (doi:10.1186/s12967-014-0308-9) contains supplementary material, which is available to authorized users.

## Background

Liver failure (LF) has a very high mortality rate due to the loss of functional liver mass below a critical level [1]. The loss of liver functions, such as detoxification, metabolic and regulatory activities, may cause severe complications, including hepatic coma, systemic hemodynamic dysfunction and multi-organ failure [1-3]. Hepatitis B related LF is the most common severe disease requiring immediate hospitalization in China [4]. Although the pathologic mechanisms underlying hepatitis B related LF are not fully understood, evidence suggests that the immune response is involved in the pathogenesis of liver injury [2].

Natural killer (NK) cells are a fundamental component of the innate immune system, and they play an important role in the first-line defense against viral infections and tumor transformation without prior sensitization [5-7]. Hepatic NK cells which represent 20%-30% of liver lymphocytes, are located in the liver sinusoids and are adherent to the endothelium [8-10]. Increasing evidence suggests that NK cells play a pivotal role in the pathogenesis of liver injury, thus contributing to LF. Hepatic NK cells can directly induce hepatocyte injury through the surface expression of death ligands (TRAIL/TRAIL receptor, Fas/Fas ligand and NKG2D/NKG2D ligand) and the release of perforin [11-14]. The production of IFN-γ and TNF-α, a hallmark of NK cell activation, is another important mechanism contributing to liver injury, which occurs through the induction of hepatocyte apoptosis and activation/recruitment of other immune effector cells [12,15,16]. However, few studies have investigated the phenotypes and functions of NK cells involved in hepatitis B related LF, and the precise mechanism underlying NK cell regulation is not fully understood.

Fibroblasts are ubiquitous cells that provide more than a source of scaffolding on which other cells function and migrate. Fibroblasts play an important role in initiating inflammation via leukocyte recruitment to the site of tissue injury [17]. Moreover, recent research has reported that fibroblasts isolated from different tumors can modulate T or NK cell functions [18,19]. Following hepatic injury, the liver stroma undergoes extensive remodeling by liver myofibroblasts (LMFs) that are principally derived from activated hepatic stellate cells (HSCs) [20,21]. LMFs can release cytokines and chemokines, such as IL-6, IL-12, HGF, VEGF and CXCL8, to promote the recruitment and positioning of lymphocytes in the inflamed liver as well as affect immune responses [22]. In a murine study, it was shown that activated HSCs attenuated intrahepatic T cell activation [23,24]. However, few studies have focused on the effect of LMFs from hepatitis B related LF patients on NK cells.

In the present study, we found that the percentage of peripheral NK cells was down-regulated with dysfunction in hepatitis B related LF patients. Our study also consistently showed that LMFs inhibited the IL-2-induced up-regulation of NK cell triggering receptors, cytokine production and cytotoxicity via prostaglandin (PG) E2 production in vitro using a cell-cell direct interaction model. Our research may provide novel insight into the pathogenesis of hepatitis B related LF.

## Methods

### Patients and specimens

Liver tissues and peripheral blood were all obtained from patients in the medical center of Sun Yat-sen University as described in our previous report [25]. Blood were from 20 patients with hepatitis B induced liver failure (Additional file 1: Table S1) and 20 healthy individuals as controls; diseased liver tissues were from 4 patients undergoing transplantation for hepatitis B induced LF (Additional file 1: Table S1); healthy livers were from 3 patients undergoing surgery for hepatic hemangioma; normal skin fibroblasts (NFs) were from 2 patients undergoing circumcision. All the samples were anonymously coded in accordance with the local ethical guidelines, as stipulated by the Declaration of Helsinki. Written informed consent was obtained from the patients, and the protocol was approved by the Review Board of Sun Yat-sen University.

### Isolation and culture of fibroblasts

NFs and LMFs were isolated as described previously [25]. Briefly, 50 grams of liver tissue or 20 grams of foreskin sample was diced and digested using type-I collagenase (100 U/mL; GIBCO, USA) and hyaluronidase (125 U/mL; Sigma-Aldrich, St. Louis, MO) followed by mechanical homogenization in a Stomacher 60 Circulator (Seward, NY, USA). The cell suspensions derived from liver specimens were cultured in DMEM medium plus 10% FBS (GIBCO, USA). Fibroblasts that had been passaged for up to 3-8 passage doublings were used for subsequent experiments to minimize clonal selection and culture stress, which can occur during extended tissue culture.

### Immunofluorescence

LMFs were cultured in 48-well flat-bottom plates. When the cells were approximately 50% confluent, they were fixed with 100% carbinol (15 minutes), rinsed and pre-wetted with phosphate-buffered saline (PBS) prior to the addition of mouse anti-human monoclonal antibodies (mAbs)-vimentin, fibronectin and α-smooth muscle actin (α-SMA), rabbit anti-human mAb-fibroblast surface protein (FSP) and immunoglobulin (IgG) controls in Tris-buffered saline (pH 7.4) for 60 minutes. The mAbs were all purchased from Abcam (Cambridge, MA, USA). The cells were washed and incubated for 30 minutes in isotype-relevant donkey anti-mouse or anti-rabbit fluorescein-isothiocyanate-conjugated secondary antibodies (IgG-AF555 or IgG-AF488, respectively; Molecular Probes, Carlsbad, CA), and the nuclei were counterstained with 40-6-diamidino-2-phenylindole (Sigma Aldrich, St. Louis, MO, USA). The images were viewed and assessed using a fluorescence microscope (LEICA DMI 4000B; Germany) at 488 nm and analyzed with Leica Application suite software (version 4.0).

### Immunohistochemistry

Paraffin-embedded samples were cut into 5 μm sections and processed for immunohistochemistry according to Kuang et al. [26]. Following incubation with anti-CD56Ab and α-SMA (Abcam, Cambridge, MA), the sections were stained using the Envision System with diaminobenzidine (DakoCytomation, Glostrup, Denmark).

### Isolation of NK cells

To obtain purified NK cells, peripheral blood mononuclear cells from healthy donors were first isolated via density gradient centrifugation, according to Pradier et al. [27], and magnetic active cell sorting (Miltenyi Biotec GmbH, Bergisch Gladbach, Germany) was subsequently used to obtain NK cells. Only the populations displaying more than 90% CD56^+^CD3^-^ NK cells were selected.

### NK cell/fibroblast co-culture

NK cells from healthy donors were cultured in RPMI-1640 plus 10% FBS in 48-well flat-bottom microtiter plates (5 × 10^4^ cells per well) in the absence or presence of fibroblasts (NK:fibroblast ratio, 2.5:1), and 100 IU/mL rhIL-2 (R&D systems, Oxford, United Kingdom) was added when indicated. At the indicated time intervals, NK cells were harvested, counted and analyzed. When indicated, 0.5 mM 1-methyl-tryptophan (Sigma, St. Louis, MO, USA) and/or 5 μM NS398 (Cayman Chemicals, Ann Arbor, MI, USA) were added at the onset of the co-cultures.

### Flow cytometry analysis

Mouse anti-human mAbs against CD73, CD56, CD69, NKp44, NKp30, NKp46, NKG2D, DNAM-1 and granzyme B were purchased from BD Biosciences (San Jose, CA, USA), mouse anti-human mAbs against CD105, CD90, CD13, CD44, CD31, CD34, CD45 and CD3 were purchased from eBioscience (San Diego, CA, USA) and mAbs against TNF-α, IFN-γ and perforin were obtained from Beckman Coulter (Fullerton, CA, USA) and Biolegend (San Diego, CA, USA).

The cells were collected, washed, and resuspended in PBS supplemented with 1% heat-inactivated FBS. Thereafter, the cells were directly stained extracellularly with 2 μg mAb in a total staining volume of 100 μL per 10^6^ cells. The cells were stimulated at 37°C for 4 hours with Leukocyte Activation Cocktail (BD Pharmingen, San Jose, CA, USA) and fixed and permeabilized prior to intracellular staining using Pharmingen's staining protocol. The data were acquired on a Gallios instrument (Beckman Coulter, Brea, CA, USA) and analyzed with FlowJo software.

### Cytotoxicity assays

The NK cells were tested for cytolytic activity via killing assays. Briefly, the purified NK cells derived from healthy donors were cultured with rhIL-2 in the presence or absence of fibroblasts. K562 cells were used as target cells (a generous gift from Dr. Dongjun Lin, Leukemia Research Institute of Sun Yat-sen University) and cocultured with NK cells for 2 hours at 37°C in 96-well V-bottom plates (2 × 10^4^ K562 cells per well). Thereafter, the target cells were stained with 5 μL annexin V (AV)-FITC and propidium iodide (PI), according to Kurschus et al. [28], and incubated in the dark for 15 minutes at room temperature. The cells were analyzed using multicolor flow cytometry (FACS Vantage-SE, BD Immunocytometry Systems, San Diego, CA, USA). Apoptotic and dead cells were identified by annexin V and PI staining. The lytic potential of the NK cells was tested by plating cells at different effector-to-target cell (E/T) ratios.

### ELISA

Supernatants were generated by seeding 5 × 10^4^ cells per well into 48-well plates in 500 μL of RPMI-1640/1% bovine serum albumin (BSA) containing 2 mmol/L L-glutamine, 60 μg/mL benzylpenicillin and 100 μg/mL streptomycin (all purchased from Sigma Aldrich, St. Louis, MO, USA). The conditioned culture supernatants were collected and analyzed for the presence of PGE2 by specific ELISA kits according to the manufacturer’s instructions (R&D systems, Abingdon, UK).

### Western blotting

The fibroblasts were cultured for 5 days in RPMI-1640 supplemented with 10% FBS and 100 U/mL rhIL-2 in 24-well cell culture cluster flat-bottom plates (2.5 × 10^5^ cells per well) in the absence or presence of NK cells (NK:fibroblast ratio, 2.5:1). Equal amounts of cellular protein were separated by 10% SDS-PAGE, immunoblotted with Abs against COX-2 (Abcam, Cambridge, MA, USA) and β-actin (Santa Cruz Biotechnology, Inc., Santa Cruz, CA, USA) and visualized with an ECL kit.

### Statistical analysis

Multiple comparisons were made between the different groups via the Mann-Whitney U test. All of the calculations were performed using Prism software (release 5.00, GraphPad Software, San Diego, CA, USA). A value of P <0.05 was considered statistically significant.

## Results

### The percentage of peripheral NK cells are decreased with dysfunction in hepatitis B related LF patients

**Figure 1:**
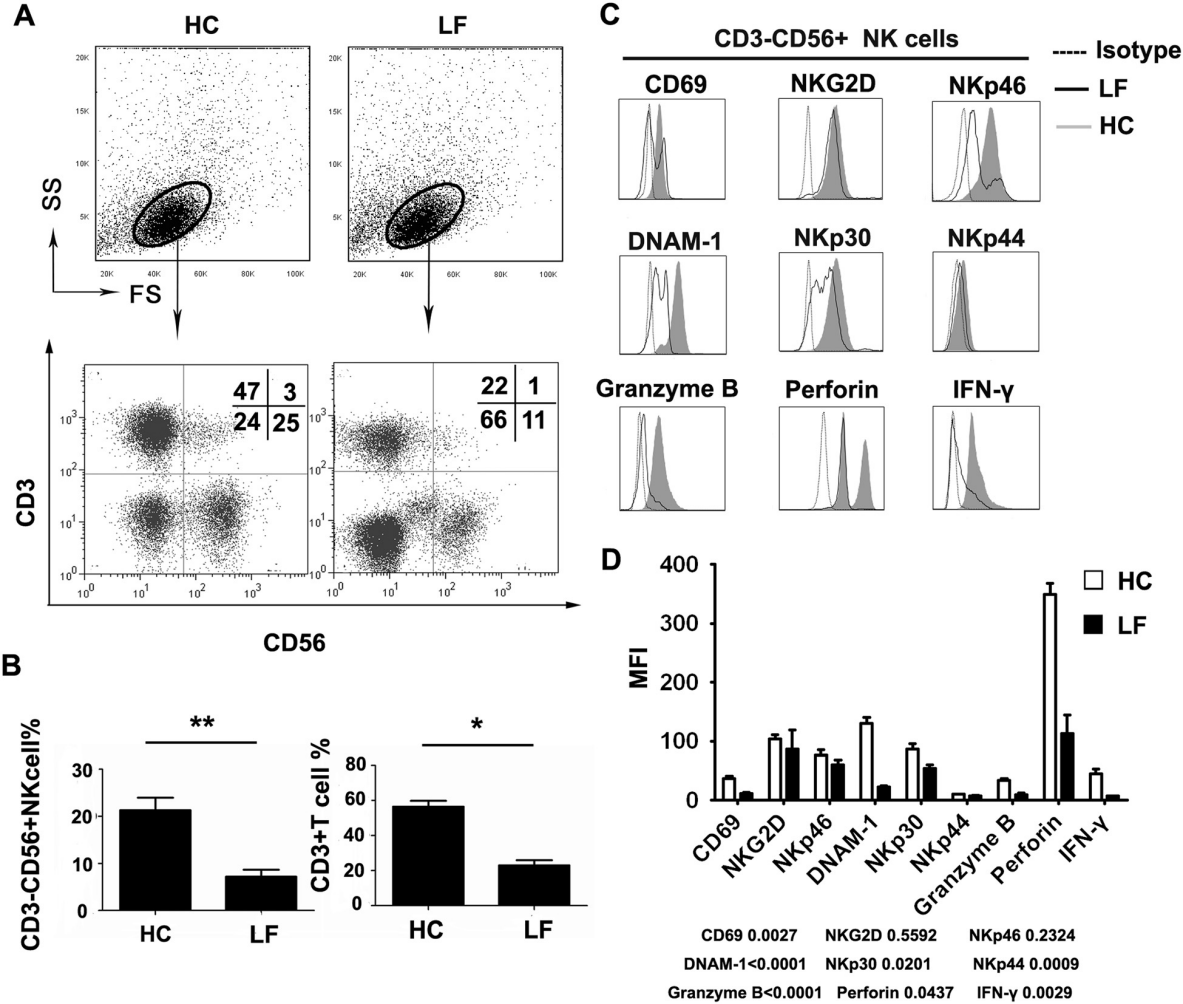
**The variation in peripheral blood NK cells between hepatitis B related LF patients and healthy controls. (A)** Representative dot plots of CD3^+^ and CD56^+^ staining in PBMCs isolated from healthy control subjects (HC) and hepatitis B related LF patients (LF). The total number of 1 × 10^5^ cells per gate was analyzed. The values in the quadrants represent the percentages of CD3^-^CD56^+^ NK cells, CD3^+^CD56^+^ NKT cells, CD3^+^CD56^-^ T cells, and CD3^-^CD56^-^ cells. **(B)** The summarized data show the percentages of CD3^-^CD56^+^ NK cells and CD3^+^ T cells in HC and LF. **(C)** CD3^-^CD56^+^ NK cells were gated. Representative primary FACS histograms depict the expression of activation markers, including NKp30, NKp44, NKp46, NKG2D, DNAM-1, CD69, cytolytic granules (perforin and granzyme B) and cytokine (IFN-γ) production by peripheral NK cells from LF (open profiles with solid lines) and HC (gray filled profiles). Open profiles with dotted lines show the isotype control. **(D)** Pooled data show the MFI of peripheral NK cells expressing activation receptors, as well as perforin, granzyme B and IFN-γ production from HC (open profiles) and LF (black profiles). The values given in **B** and **D** represent the means ± SEM of five separate experiments. *P <0.05 and **P <0.01 indicate significant differences between HC and LF **(B and D)**.

To better understand the potential role of NK cells in the pathogenesis of hepatitis B related LF, we first detected the distribution and phenotype of peripheral NK cells in these patients. We dissected the subtypes of peripheral blood leukocytes in 20 hepatitis B related LF patients and 20 healthy controls by flow cytometry analysis for CD56^+^CD3^-^ NK cells and CD3^+^ T cells. In comparison with healthy control subjects, the percentages of NK cells and CD3^+^ T cells were significantly reduced in hepatitis B related LF patients (Figure 1A and B). We further analyzed the expression of NK activation markers, including NKp30, NKp44, NKp46, NKG2D, DNAM-1, CD69, cytolytic granules (perforin and granzyme B) and cytokine (IFN-γ) production by NK cells in the two groups of individuals. As illustrated in Figure 1C and D, the expression of most of the activation markers was down-regulated except for NKG2D and NKp46, and cytolytic granules and IFN-γ production were inhibited in the peripheral NK cells of hepatitis B related LF patients, suggesting their dysfunctional status.

### LMFs are in close proximity to NK cells in liver tissue of hepatitis B related LF patients

**Figure 2:**
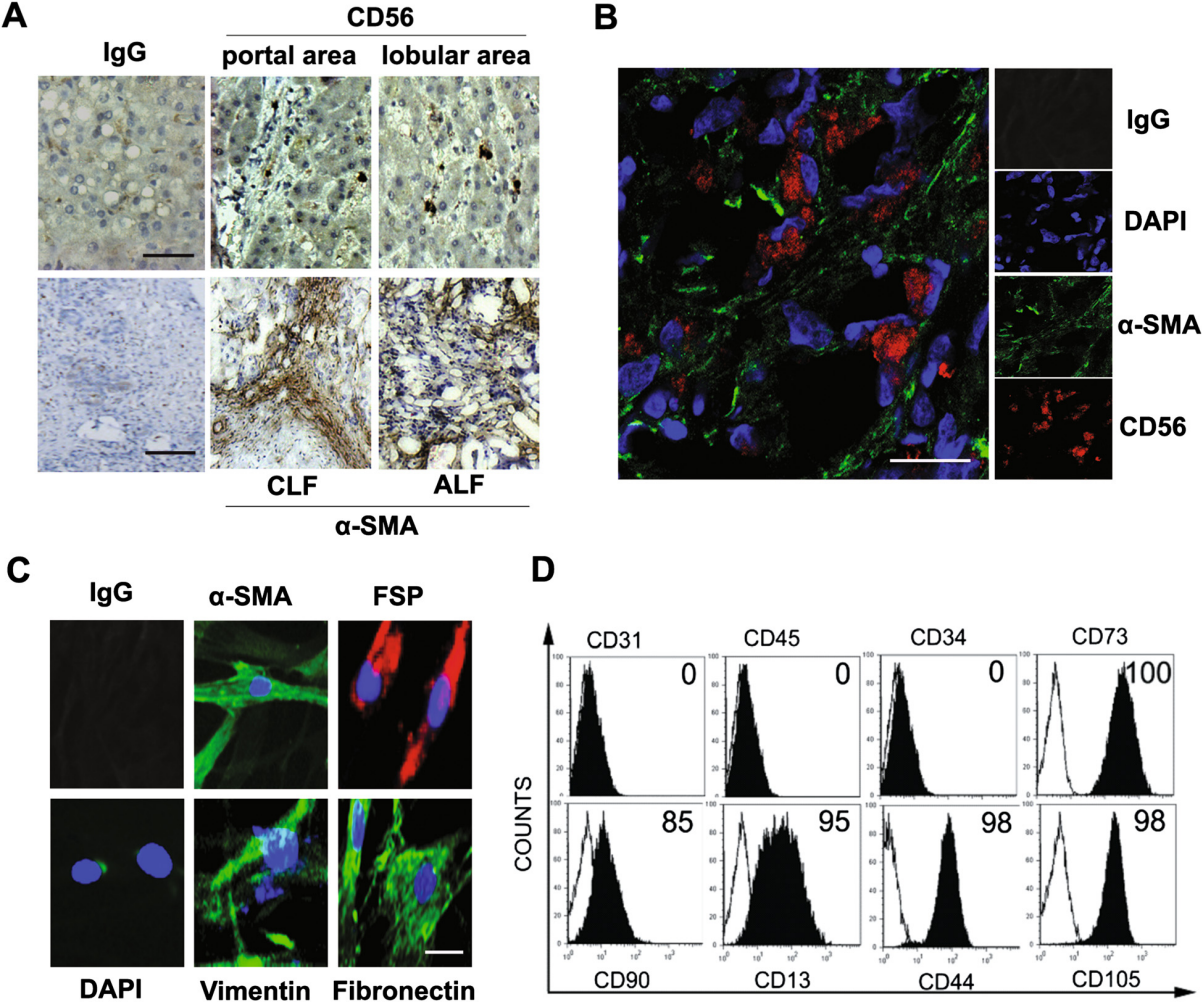
**Co-localization of LMFs and NK cells in hepatitis B related LF patients and phenotypic characterization of LMFs. (A)** CD56^+^ NK cells infiltrate the diseased liver tissues (bar, 100 μm), and a high level of α-SMA is expressed in both chronic (CLF) and acute (ALF) hepatitis B related LF patients (bar, 200 μm). 1 of 15 representative micrographs is shown. **(B)** Confocal immunofluorescence shows that CD56^+^ NK cells are in close proximity to LMFs (α-SMA^+^) at the sites of liver inflammation, and 1 of 15 representative micrographs is shown. Bar, 50 μm. **(C)** Immunofluorescence staining of LMFs isolated from representative samples of LF patients. Bar, 20 μm. **(D)** The surface markers of LMFs cultured for 3-5 population doublings were analyzed by flow cytometry (open profiles, isotype; black filled profiles, stained with indicated Abs). The percentage of positive cells is shown.

During chronic liver injury, LMFs contribute to tissue repair alongside regions of cellular damage [29]. However, it is unclear whether LMFs play a role in acute liver injury. As shown in Figure 2A, the α-SMA expression was detectable in patients with acute hepatitis B related LF, suggesting the same physiologic response of LMFs during different conditions of liver injury. To further elucidate the roles of LMFs in hepatitis B related LF, we examined the infiltration of hepatic CD56^+^ NK cells and α-SMA^+^ LMFs (Figure 2A and B). Interestingly, the NK cells colocalized with the LMFs at sites of liver inflammation (Figure 2B).

### The characteristics of LMFs

To determine the specific effect of LMFs on NK cells, we first established highly purified subsets of LMFs. The LMFs were established from the liver tissues of 4 hepatitis B related LF patients as mentioned above in material section. The phenotypes of the isolated LMFs were analyzed by immunofluorescence and flow cytometry. The cultures were of high purity, had the characteristic spindle-shape, and expressed fibroblast markers (α-SMA, vimentin, FSP, and fibronectin; Figure 2C). We subsequently used flow cytometry to determine the purity of LMFs cultured in vitro. As shown in Figure 2D, all the LMFs expressed fibroblast markers (CD73, CD90, CD13, CD44 and CD105) and failed to express endothelial, hematopoietic or epithelial cell markers (CD31, CD45 and CD34).

### LMFs trigger NK cell dysfunction in vitro

**Figure 3:**
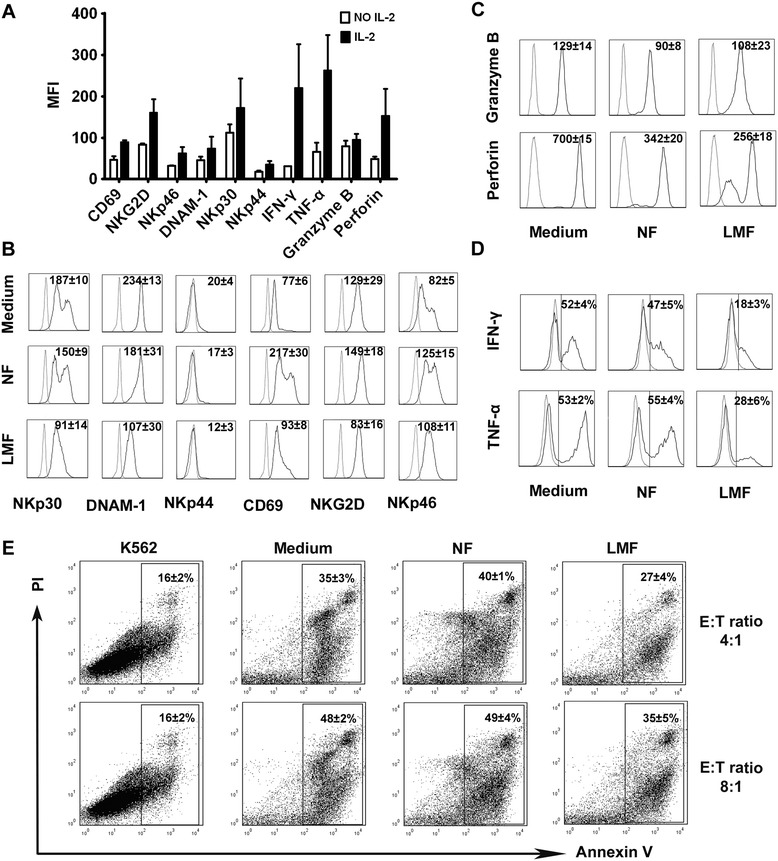
**LMFs regulate NK cell function.** NK cells were cultured in rhIL-2 alone or with the indicated fibroblast cells and analyzed by flow cytometry. **(A)** After 5 days of culture in the absence or presence of rhIL-2, the triggering receptors, cytolytic granules and cytokine production of NK cells were analyzed. **(B)** The expression of NK cell triggering receptors was analyzed. **(C)** Intracytoplasmic analysis of granzyme B and perforin expression. **(D)** Analysis of the production of IFN-γ and TNF-α by NK cells under different conditions. **(E)** LMF-conditioned NK cells showed reduced cytotoxicity against K562 cells at different T/E ratios. The mean fluorescence intensities (MFI; indicated as the mean ± SEM of 7 independent experiments; **A**, **B** and **C**) and the percentages of positive cells **(D)** or apoptotic cells **(E)** are shown. The open profiles with dotted lines show the isotype control, and the open profiles with solid lines show the expression of the indicated markers.

We next sought to determine whether LMFs were able to regulate NK cell phenotypes and functions. NK cells freshly isolated from the circulation of healthy donors were cultured in the absence or presence of rhIL-2. After 5 days of culture, the expression of NK cell triggering receptors (including NKp46, NKp30, NKp44, NKG2D and DNAM-1) and the CD69 activation marker was significantly up-regulated in the presence of rhIL-2 (Figure 3A). In addition, the release of cytolytic granules (perforin and granzyme B) and the secretion of cytokines (TNF-α and IFN-γ) were up-regulated by rhIL-2 treatment (Figure 3A).

To determine the possible effects of LMFs on NK cells, mixed cell cultures were set up. Because LMFs do not exist in healthy livers, and primary HSCs undergo rapid activation in in vitro culture conditions, we used NFs as controls as used by other investigators [22]. Notably, LMFs significantly inhibited the rhIL-2-induced up-regulation of NKp30, DNAM-1, NKp44 and NKG2D receptors, while no inhibitory effect could be detected on NKp46 and CD69. In contrast, NK cells had only a marginal decrease in the expression of NKp30 and DNAM-1 upon co-culture with NFs (Figure 3B). Interestingly, the LMFs significantly inhibited the rhIL-2-induced up-regulation of perforin, while the effect on granzyme B secretion was slight (Figure 3C). Moreover, the LMFs significantly interfered with the NK cell secretion of TNF-α and IFN-γ, while the NFs had almost no effect (Figure 3D).

The down-regulation of perforin and granzyme B expression in NK cells exposed to LMFs suggests an impairment in NK cytotoxicity. Therefore, our next endeavor was to determine the lytic potential of NK cells in response to LMFs. Purified NK cells (Medium) or NK cells that had been cultured with LMFs (LMF) or NFs (NF) were incubated with K562 cells in the presence of rhIL-2 at various effector-to-target cell ratios. Consistent with our hypothesis, as shown in Figure 3E, the cytotoxicity of the NK cells against K562 cells was remarkably attenuated upon co-culture with LMFs (E/T = 4:1, 35 ± 3 vs. 27 ± 4; E/T = 8:1, 48 ± 2 vs. 35 ± 5). In contrast, the co-culture with NFs had no effect on the NK cytotoxicity.

### Both cell-cell interactions and soluble factor(s) are involved in the inhibitory effect of LMFs on NK cells

In an attempt to investigate whether the cell-cell contact between NK cells and LMFs was responsible for the inhibitory effects of LMFs, co-culture experiments were performed in transwells. As shown in Additional file 2: Figure S1, the inhibitory effect of LMFs on the expression of NKp30 under transwell conditions was still apparent, although reduced, while the expression of DNAM-1 and NKG2D was minimally affected. These results suggest that LMFs constitutively release soluble factor(s) capable of interfering with NKp30 up-regulation. Such release would be significantly enhanced by the direct interaction between LMFs and NK cells. In contrast, the inhibition of DNAM-1 and NKG2D expression appears to be dependent on cell-cell contact.

### PGE2 release is involved in the inhibitory effect of LMFs on NK cells

**Figure 4:**
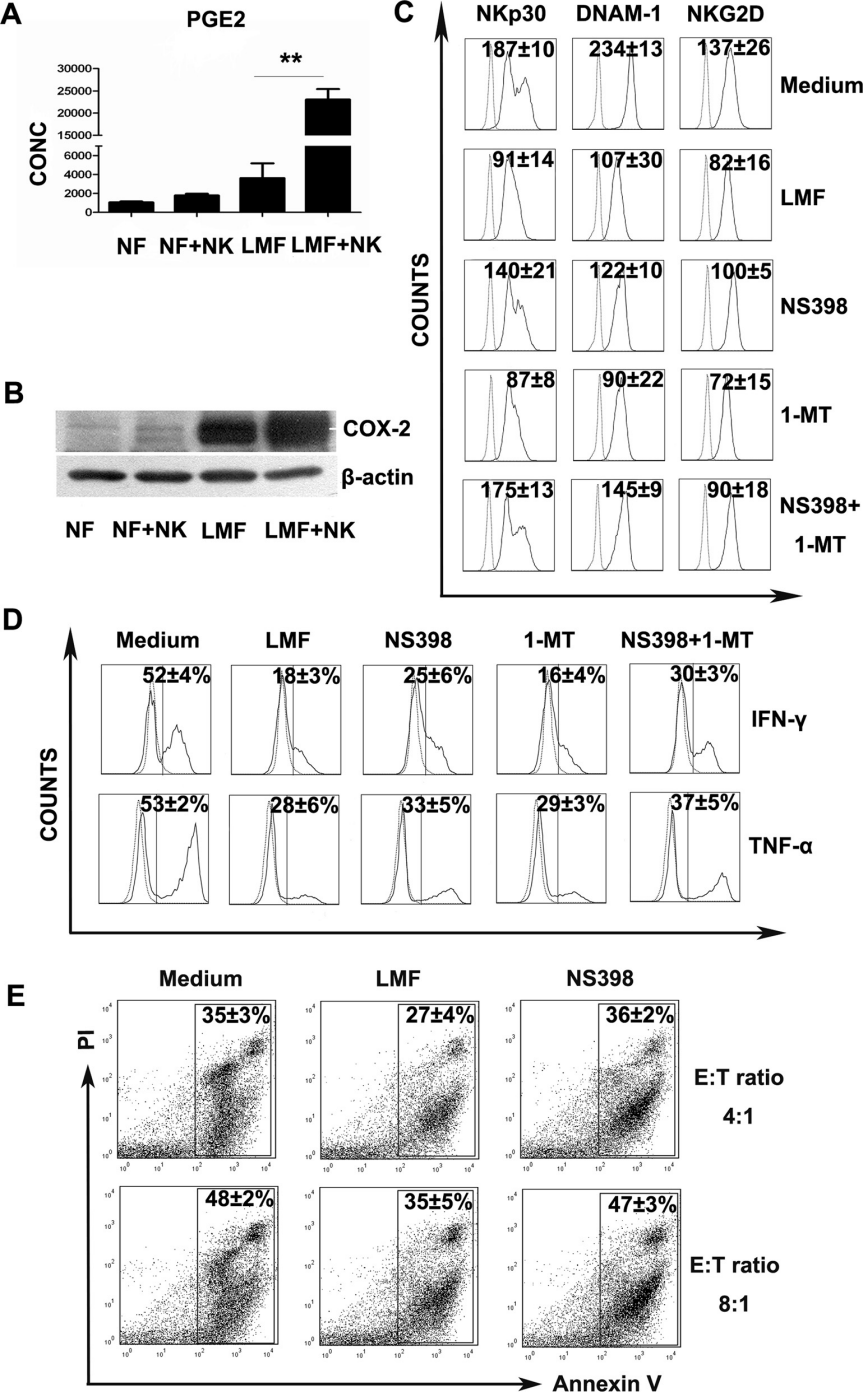
**Role of PGE2 in the immunoregulatory effect of LMFs on NK cells.** NK cells were co-cultured with LMFs in the absence or presence of the indicated inhibitors for 5 days with rhIL-2. **(A)** NK cells were left untreated except for rhIL-2 (NK) or were cultured with the indicated fibroblasts (NF, normal skin fibroblasts; LMF, LMFs) for 5 days. The concentrations of PGE2 (pg/mL) in the supernatants were assessed by ELISA. **(B)** The expression of COX-2 by the above-mentioned cells were assessed by Western blotting. **(C)** The variations in NKp30, DNAM-1 and NKG2D in the presence of the indicated inhibitors. **(D)** The secretion of IFN-γ and TNF-α by NK cells was affected by inhibitors. **(E)** LMF-conditioned NK cells showed restored cytotoxicity against K562 cells with the inhibitors. The mean fluorescence intensities (MFI; indicated as the mean ± SEM of 7 independent experiments) **(C)** and the percentages of receptor-positive cells **(D)** or apoptotic cells **(E)** are shown. The open profiles with dotted lines show the isotype control, and the open profiles with solid lines show the expression of the indicated markers. *P<0.05, **P<0.01.

It has been reported that PGE2 is the primary fibroblast-derived immunoregulatory factor [19]. To determine the role of PGE2 in the NK/LMF co-culture system, culture supernatants were harvested, and the concentrations of PGE2 were measured by ELISA. As shown in Figure 4A, the supernatants derived from LMFs alone contained PGE2, indicating their spontaneous release. Importantly, PGE2 release by LMFs was greatly enhanced in the presence of NK cells (Figure 4A), which suggests that bidirectional communication between LMFs and NK cells may contribute to the increased release of PGE2. Subsequently, LMF proteins were harvested and analyzed by Western blotting for cyclooxygenase 2 (COX-2), which is an inducible enzyme involved in the production of PGs during inflammation. Consistent with the above results, LMFs spontaneously released large amounts of COX-2, while NFs produced low levels of COX-2. After co-culture with NK cells, the production of COX-2 by LMFs was markedly increased (Figure 4B).

To further confirm the effect of PGE2 on NK cells, co-culture experiments were performed in the presence or absence of the PGE2 inhibitor NS398. The addition of NS398 to the co-culture largely restored the expression of NKp30, DNAM-1 and NKG2D, and the secretion of IFN-γ and TNF-α was partially restored (Figure 4C and D). In contrast, the IDO inhibitor, 1-methyl-tryptophan (1MT), had almost no effect on the restoration of NK cell dysfunction (a slight effect was detected only when used in combination with NS398). Consistent with these findings, cytotoxicity assays showed that blocking PGE2 largely restored the ability of NK cells to induce the apoptosis of K562 cells (Figure 4E).

## Discussion

In the present study, we provide evidence that LMFs isolated from hepatitis B related LF patients interfere substantially with the activation of NK cells in vitro. We show that LMFs attenuated rhIL-2-induced NK cell triggering receptors, cytotoxicity and cytokine production. This suppressive function, which is largely dependent on PGE2 release, appears to be specifically induced by LMFs; NFs had a minimal effect on NK cells and released low levels of PGE2.

In the current study, we observed that NKp30 were expressed at lower levels on peripheral NK cells in hepatitis B related LF patients compared to healthy controls (P = 0.0201). However, Zou Y et al. reported up-regulated NKp30 expression on peripheral NK cells in patients with HBV-related acute-on-chronic LF [12]. The controversy regarding NKp30 expression on NK cells of LF patients may derive from the use of different controls and the complexity of the disease. Firstly, the controls in our study were all from healthy donors, but in the report by Zou Y et al., all the controls were from chronic hepatitis B patients. Secondly, all the samples in our study were from patients with late stage LF, which means that the patients were preparing for liver transplantation. However, in the report by Zou Y et al., the conditions of the patients appeared improved, and they may have recovered as a result of conservative treatments. We speculate that at the early stage of liver injury, NK cells may be activated and accelerate inflammation, but at the late stage of liver disease, the function of NK cells is suppressed. This notion is also supported by evidence that the cytolytic activities of NK cells increase during HBV-related spontaneous “hepatic flares” (the early stage of liver injury) [14,30] and that NK cell functions are suppressed during advanced liver injury [31].

We studied differentiated LMFs isolated directly from different diseased human livers. There were no consistent differences between the LMFs isolated from patients with hepatitis B related LF, and all the LMFs expressed similar markers and could secrete similar levels of PGE2 (data not shown). Interestingly, the HSCs from healthy livers could also be activated into LMFs through in vitro culture, and no differences were found in their levels of PGE2 secretion (Additional file 3: Figure S2), suggesting their stability for further research. Consistent with our results, other investigators have recently reported that LMF preparations from diseased livers of different etiologies secreted similar patterns of proinflammatory cytokines and chemokines [22].

The present investigation provides evidence that LMFs can regulate NK cell activation in vitro. Hepatic NK cells are comparable to in vitro IL-2-activated NK cells derived from the spleen or blood and are thus considered to be naturally activated by the hepatic microenvironment [32]. Therefore, we used IL-2-stimulated NK cells isolated from the peripheral blood of healthy donors for our cultures. With respect to the mechanisms of inhibition, we showed that the NK cell activation receptors, including NKp30, DNAM-1, NKp44 and NKG2D, were partially inhibited when exposed to LMFs. Indeed, the surface expression of CD69 and NKp46 was not inhibited upon LMF co-culture in the study. We believe that the effect of LMFs on NK cells did not involve a general blockade of NK cell activation. Consistent with our findings, investigators have reported that the surface expression of CD69 and NKp46 that are either up-regulated (NKp46) or induced ex-novo (CD69) after exposure to IL-2 was not inhibited by the interaction with tumor-associated fibroblasts [19]. Elucidating the molecular mechanisms of action by LMFs on NK cells is an interesting and ongoing research project in our laboratory.

Under co-culture conditions, NFs, which don’t release PGE2, also inhibit the expression of NKp30 and DNAM-1 on NK cells (albeit to a lesser extent). This was consistent with previous observations and suggested that fibroblasts, which are rather numerous in organisms, may represent a cell population that is underrated for its involvement in immunomodulation [33]. In a recent study, we observed that NFs could also release different soluble factors [34]. We accept that it is highly likely that other soluble factors may also be responsible for fibroblast-induced immunosuppression on NK cells. However, we argue that PGE2 is likely to represent a driver of inhibition on NK cells.

In the current study, we observed that LMFs produced more PGE2 when they were in contact with NK cells, which in turn led to dysfunctional NK cells. These data suggest a negative feedback mechanism involving the LMF-PGE2-NK cell axis. The downregulation of NK cell function during liver injury may facilitate liver regeneration and prevent further hepatocyte injury [10]. However, accumulating evidence supports an antifibrotic role for NK cells via the inhibition of HSCs by apoptosis and IFN-γ production [35-37]. Therefore, the dysfunction of NK cells during liver injury may facilitate liver fibrosis or cirrhosis [38]. Simultaneously, decreased NK cell activity in cirrhotic patients has been related to an increased incidence and invasiveness of hepatocellular carcinoma [38,39]. The tumor cells may escape from NK cell immune surveillance and directly and indirectly facilitate tumor progression and metastasis [40]. Therefore, we believe that LMFs are double-edged swords.

The precise mechanisms underlying the deterioration of liver function during hepatitis B related LF remain unclear. However, the impairment of cellular immunity is believed to be a contributing factor. In general, activated NK cells accelerate liver injury by producing proinflammatory cytokines, such as granzyme B and IFN-γ, and by killing hepatocytes [41]. Moreover, the function of NK cells can be further enhanced by other cytokines, such as IFN-γ, IL-8, IL-12 and IL-15 [14,30]. The induction of PGE2 by LMFs may represent a mechanism of limiting liver damage, such that few patients with fibrosis develop fulminant LF [42]. Consistent with our results, a recent study reports that liver fibrosis may be protective in the context of acute liver injury [43]. We hypothesize that NK cells may be inhibited and preferentially skewed toward suppression activity during hepatitis B related LF, depending on the balance between the levels of PGE2 and cytokines mentioned above. Further investigation is therefore warranted to determine the hypothesis.

Our study has limitations. First, most of our experiments were done in vitro, which may not stand for the in vivo state of hepatitis B related LF patients. Nevertheless, our study is of hypothesis-generating value for the design of future in vivo studies. Second, the 20 patients from whom serum was studied and the 4 liver tissues of subjects with hepatitis B related LF are not representative for all phases of LF and these numbers are small with respect to the different conditions of these LF patients. In China, hepatitis B is involved in more than 80% of LF cases due to a high incidence of HBV infection, and most LF patients died except for liver transplantation [44]. However it is very difficult to obtain sufficient quantities of liver tissue from these kind of patients for the shortage of donor organs. Nonetheless, since there were no consistent differences that characterized LMFs isolated from different hepatitis B related LF patients in our study, we believe that this phenomenon is not peculiar to a few patients and as seems more likely applicable to most LF patients. Despite these shortcomings, we believe our analysis shows the role of LMFs in the regulation of NK cell functions during the process of hepatitis B related LF. Larger studies are necessary to fully address this important question.

In conclusion, LMFs not only regulate remodeling against liver injury by producing the extracellular matrix but also release PGE2, which can regulate immune responses. The notion that NK cells can trigger a paradoxical suppressive loop by enhancing the release of PGE2 by LMFs in vitro reveals an interesting area of investigation for the field of immune subversion in hepatitis B related LF patients. Indeed, this observation suggests that LMFs not only suppress NK cells but may also interact with them, thereby eliciting reciprocal cross-talk to protect hepatocytes from further injury. Further studies should be performed to extend our knowledge on this matter in view of the attempts to exploit NK cell-based cellular therapies [45].

## Additional files

Additional file 1: Table S1. Basic clinical characteristics of the patients. Additional file 2: Figure S1. LMFs also regulate NK cell function under transwell conditions. NK cells purified from healthy PBMCs were cultured with the indicated fibroblast cells in a 24-well transwell plate system (0.22 μm pore size; Costar) for 5 days. The expression of NK cell triggering receptors was analyzed by flow cytometry. The mean fluorescence intensities (MFI; indicated as the mean ± SEM of 7 independent experiments) are shown. The open profiles with dotted lines show the isotype control, and the open profiles with solid lines show the expression of the indicated markers. Additional file 3: Figure S2. The concentrations of PGE2 (pg/mL) in the supernatants of LMFs from patients (LMF) and healthy controls (NL) were assessed by ELISA.

## Abbreviations

α-SMA: α-smooth muscle actin
COX-2: Cyclooxygenase 2
FSP: Fibroblast surface protein
IDO: Indoleamine 2, 3-dioxygenase
LF: Liver failure
LMFs: Liver myofibroblasts
1MT: 1-methyl-tryptophan
NCRs: Natural cytotoxicity receptors
NFs: Normal skin fibroblasts
NK: Natural killer
PBMCs: Peripheral blood mononuclear cells
PGE2: Prostaglandins E2
