# Supplementary figures and images for: Liver myofibroblasts from hepatitis B related liver failure patients may regulate natural killer cell function via PGE2
Source: J Transl Med. 2014 Nov 4;12:308. doi: 10.1186/s12967-014-0308-9 (PMC4232720; doi:10.1186/s12967-014-0308-9)

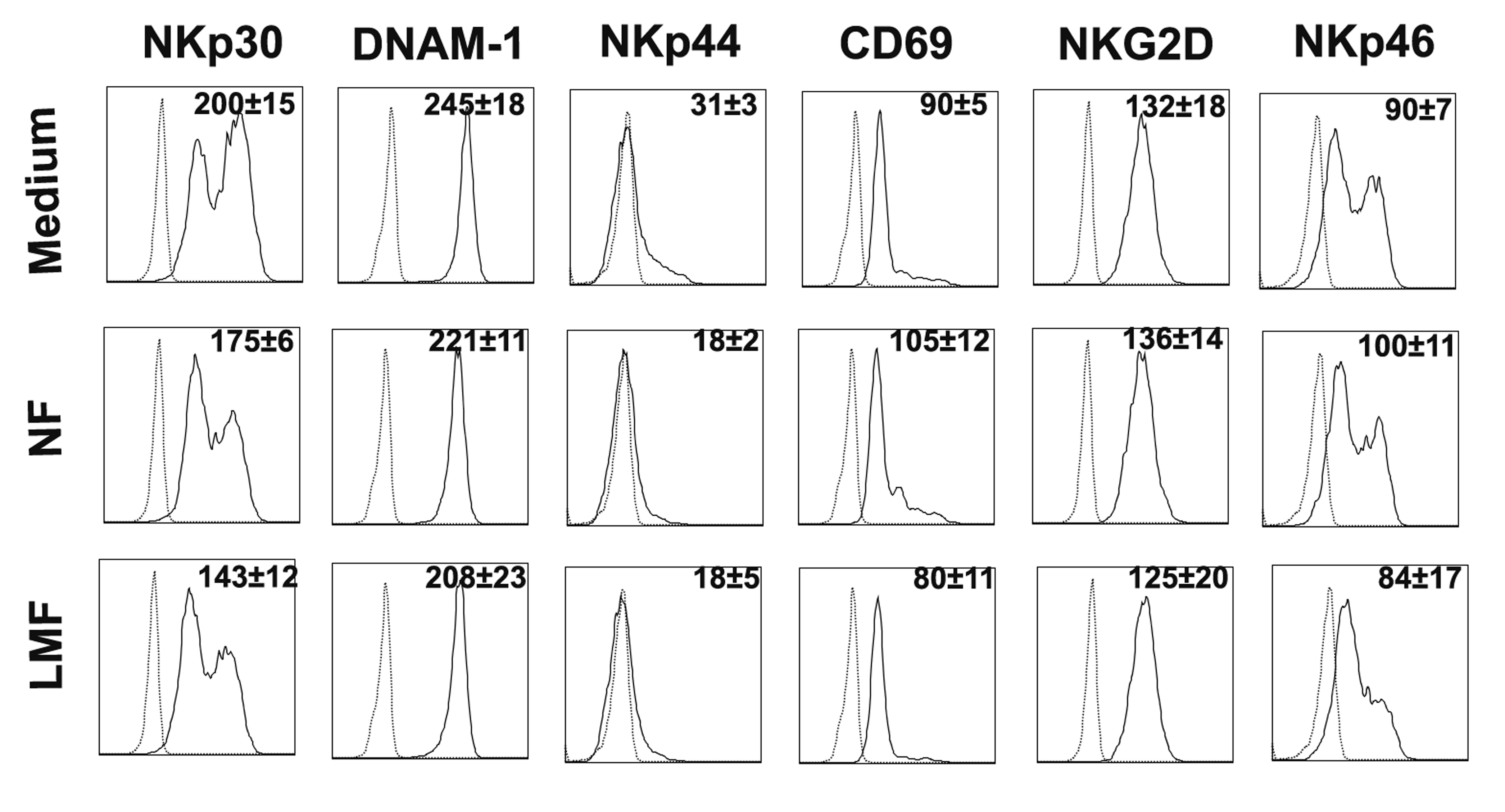

Supplement: Additional file 2: Figure S1. — LMFs also regulate NK cell function under transwell conditions. NK cells purified from healthy PBMCs were cultured with the indicated fibroblast cells in a 24-well transwell plate system (0.22 μm pore size; Costar) for 5 days. The expression of NK cell triggering receptors was analyzed by flow cytometry. The mean fluorescence intensities (MFI; indicated as the mean ± SEM of 7 independent experiments) are shown. The open profiles with dotted lines show the isotype control, and the open profiles with solid lines show the expression of the indicated markers. [file 12967_2014_308_MOESM2_ESM.tiff]

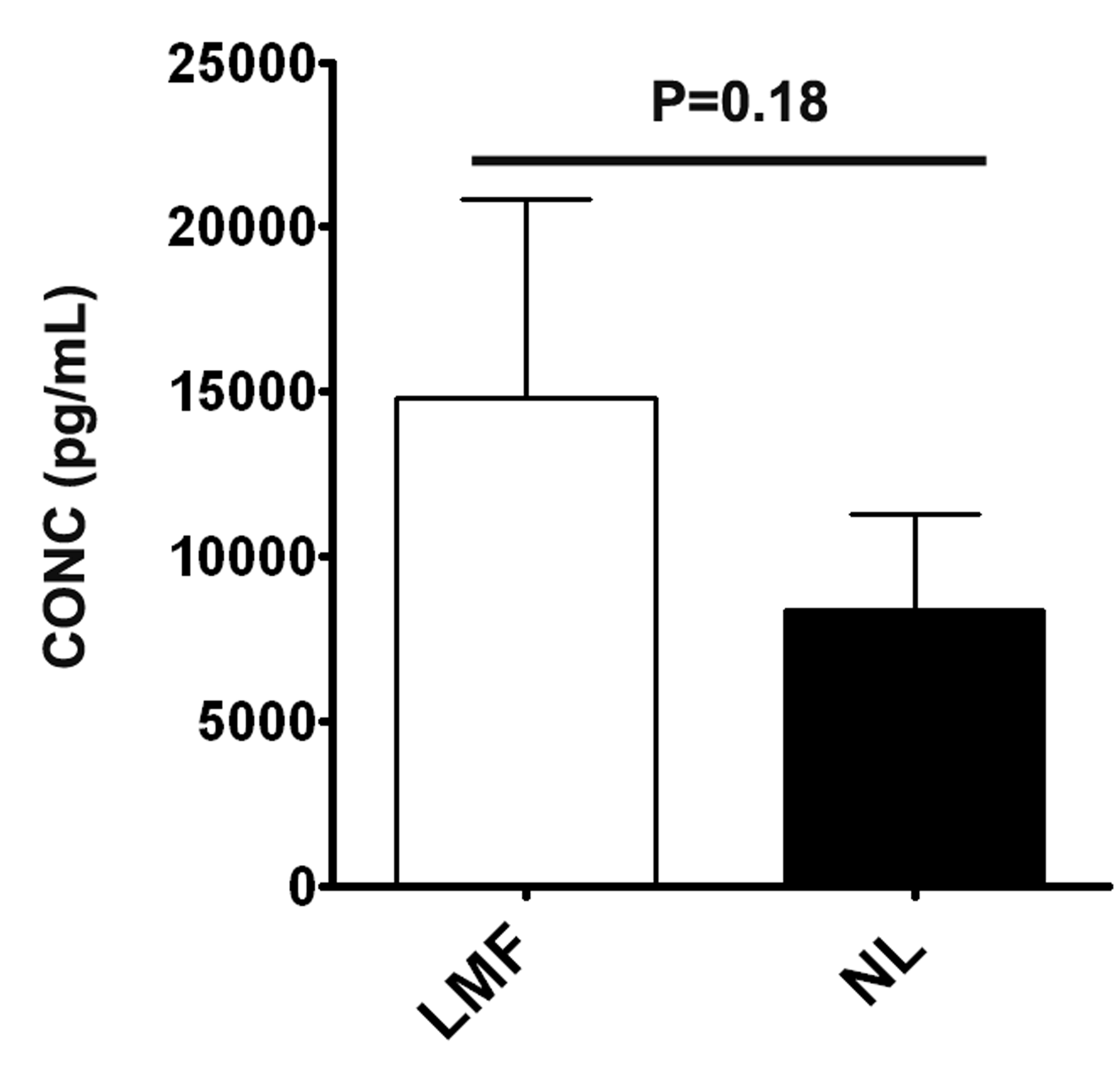

Supplement: Additional file 3: Figure S2. — The concentrations of PGE2 (pg/mL) in the supernatants of LMFs from patients (LMF) and healthy controls (NL) were assessed by ELISA. [file 12967_2014_308_MOESM3_ESM.tiff]
